# Translating physical stress into biological signals: precision electro-oncology based on conformational changes of electro-sensitive receptors and parameterized immune remodeling

**DOI:** 10.3389/fonc.2026.1854341

**Published:** 2026-07-09

**Authors:** Wei Shi, Mengya Zhao, Xiaofeng Ma, Li Dong, Yulong Sun

**Affiliations:** 1 Department of Urology, The Second Hospital of Lanzhou University, and Gansu Province Clinical Research Center for Urinary System Disease, Lanzhou, China; 2 School of Life Science and Technology, Key Laboratory for Space Biosciences & Biotechnology, Institute of Special Environmental Biophysics, Research Center of Special Environmental Biomechanics and Medical Engineering, Engineering Research Center of Chinese Ministry of Education for Biological Diagnosis, Treatment and Protection Technology and Equipment, Northwestern Polytechnical University, Xi’an, Shaanxi, China; 3 Emergency Center, The Second Hospital of Lanzhou University, Lanzhou, Gansu, China; 4 NHC Key Laboratory of Diagnosis and Therapy of Gastrointestinal Tumor, Gansu Provincial Hospital, Lanzhou, China

**Keywords:** tumor-treating fields, physical signal, bioelectric signals, electro-sensitive receptors, tumor immune microenvironment

## Abstract

Tumor Treating Fields (TTFields) and pulsed electric fields (PEF) have emerged as a fourth modality in oncological treatment, succeeding surgery, radiotherapy, and pharmacotherapy. However, the fundamental mechanisms through which physical electrical stress is precisely transduced into biochemical signals remain to be fully elucidated. By integrating molecular dynamics simulations, multi-omics profiling, and the latest clinical trial data, this review systematically explores how electric fields, functioning as physical signals, initiate intracellular transduction by inducing conformational kinetic changes in G protein-coupled receptors (GPCRs), such as NPFFR2. Furthermore, we discuss how the modulation of waveform parameters enables the precise programming of apoptosis and pyroptosis patterns, thereby inducing immunogenic cell death (ICD) and activating the cGAS-STING pathway. Additionally, proteomics-based insights are employed to reveal the mechanisms of electric-field-induced compensatory resistance, and the pivotal role of electric fields in remodeling the tumor immune microenvironment and reversing resistance to immune checkpoint inhibitors is discussed. This review aims to provide refined theoretical guidance for the clinical translation of the emerging field of the “electro-immunome”.

## Graphical Abstract

**Figure:**
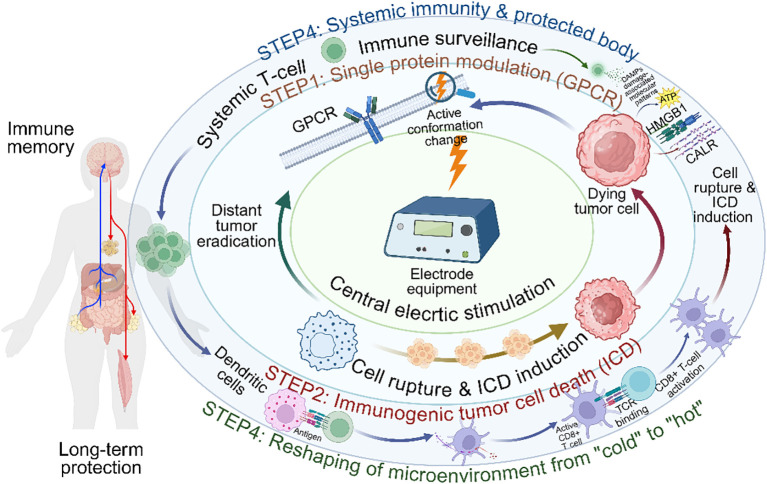
The cascade from physical perception to immune programming. This graphical abstract elucidates the multi-scale biological impact of bioelectric signals: (1) Molecular perception of electric fields through GPCRs and the cytoskeleton; (2) Programmed cell fate determination between apoptosis and pyroptosis; (3) Systemic remodeling of the tumor microenvironment from “cold” to “hot”; (4) Synergistic integration with immunotherapy to overcome acquired resistance.

## Highlights

- EFs trigger signaling via GPCR conformational kinetic changes (e.g., NPFFR2).
- Waveform parameters selectively drive cell death toward apoptosis or pyroptosis.
- Electro-stress activates cGAS-STING, shifting the tumor to a "hot" state.
- Multi-omics identifies PARP1 and BRD4 as compensatory nodes in electro-stress.
- Precision electro-oncology helps reverse immune checkpoint resistance.

## 1 Introduction

### 1.1 Evolution of tumor electric field therapy: from physical interference to biological signal transduction

In the clinical management of refractory tumors such as glioblastoma (GBM) and non-small cell lung cancer (NSCLC), the application of physical therapeutic modalities indicates an evolving direction in clinical practice (1, 2). Conventional perspectives tend to view Tumor Treating Fields (TTFields) as a purely anti-mitotic tool that disrupts microtubule protein and spindle formation to arrest cell division (3, 4). However, emerging evidence suggests that the effects of electric fields may possess a complexity far exceeding simple biophysical interference. As a physical stress signal, electric fields can be “captured” by specific structures on the cell surface and transduced into complex biological responses (5, 6). This shift in perception, moving from physical perturbation toward molecular signal transduction mechanisms, provides a theoretical foundation for the establishment of precision electro-biology.

The application of physical electrical modalities in oncology represents a significant trajectory in translational research. Historically, foundational literature predominantly focused on the dielectric polarization of tubulin during mitosis as the primary mechanism of growth inhibition (7). However, with the progressive convergence of bioelectromagnetics and tumor immunology, an increasing number of independent studies indicate that the plasma membrane and its resident transmembrane domains are sensitive to exogenous electric fields (8, 9). By expanding the focus from macromolecular mitotic interference to sub-microscopic transmembrane signal transduction and systemic immune remodeling, the framework of precision electro-oncology can be evaluated across a broader multidisciplinary literature base (10). This integration of interdisciplinary data provides an objective theoretical foundation for investigating the complex biological effects induced by physical interventions.

### 1.2 Membrane protein conformational sensing: the initial stage of electrical stress capture

The plasma membrane serves as the primary barrier for electric signal transmission, with its constituent membrane proteins hypothesized to function as “molecular antennas”. Recent studies utilizing molecular dynamics simulations have explored the direct regulatory effects of direct current electric fields (dcEF) on the conformation of G protein-coupled receptors (GPCRs). Using the NPFFR2 receptor as a model, electric fields have been shown to not only inhibit its expression in macrophages but also to alter the transmembrane kinetics of the receptor protein. This suggests that electric fields may induce allosteric effects by altering the force balance on charged amino acid residues (5). Furthermore, electric-field-induced localized rupture of the nuclear envelope leads to the release of micronuclei into the cytoplasm, activating the intracellular DNA sensors cGAS and AIM2. Such findings indicate that physical electric fields can directly initiate innate immune sensing pathways (11).

### 1.3 Immunophysical programming: cell death patterns and microenvironment remodeling

The efficacy of electric field therapy is contingent not only on field intensity but is also closely linked to the parameterized encoding of waveforms. Research suggests that precise modulation of pulse width and frequency allows for the spatial regulation of cell death pathways. For instance, specific parameter combinations can induce pro-inflammatory pyroptosis rather than silent apoptosis, leading to the significant release of damage-associated molecular patterns (DAMPs) such as HMGB1 and ATP (3, 12). This “immunophysical programming” effectively promotes the maturation of dendritic cells (DCs) and the infiltration of T cells, facilitating the conversion of “cold tumors”—previously unresponsive to immunotherapy—into “hot tumors” (13, 14). Systematic remodeling of the microenvironment has been preliminarily validated in clinical studies such as LUNAR and METIS, where the synergistic effect of electric fields and immune checkpoint inhibitors (ICI) significantly improved patient survival outcomes (1, 2).

### 1.4 Resistance challenges and clinical prospects in the multi-omics era

While electric field therapy demonstrates promising application potential, the adaptive evolution of tumor cells under electrical stress exposure warrants consideration. Multi-omics profiling reveals that chronic electric field intervention may induce compensatory upregulation of proteins such as PARP1 and BRD4, which is considered a potential mechanism for adaptive resistance (6). Additionally, the regulatory effects of electric fields on cell surface immune checkpoint receptors (e.g., PD-1, MHC-II) in melanoma and other cancers highlight the unique potential of electric fields in reversing acquired resistance (15, 16). This review aims to integrate the latest findings in molecular sensing, physical programming, and omic evolution to explore how refined electrical stimulation protocols might overcome current limitations in cancer treatment, while providing perspectives on future directions such as spatial transcriptomics and individualized array design.

However, it is pertinent to note that the establishment of the precision electro-oncology framework remains in its exploratory stages (17). Although biophysical simulations and certain preclinical models have provided heuristic insights into potential regulatory pathways (18), critical junctures still present substantial mechanistic uncertainties. These include the direct *in situ* verification of membrane receptor conformational sensing, the parameterized manipulation of non-linear cell death modes, and the systemic immune translation within complex *in vivo* microenvironments (19). Consequently, while synthesizing these advancements, this review aims to maintain a cautious scientific perspective, deliberately distinguishing between experimentally supported biological phenomena, evolving mechanistic hypotheses, and speculative directions, thereby offering an objective theoretical reference for this interdisciplinary field.

### 1.5 Review methodology and search strategy

To ensure the comprehensive nature of this synthesis and mitigate potential selection bias, a standardized literature retrieval and screening methodology was implemented. The conceptual framework of this review was established by systematically consulting three primary academic databases: PubMed, Web of Science (Core Collection), and Google Scholar. The temporal scope for literature inclusion extended from the inception of each database up to 2026, capturing recent breakthroughs at the interface of oncology and bioelectromagnetics.

The database queries utilized an interdisciplinary Boolean matrix combining biophysical and immunological domains. The primary keyword combinations included: (1) physical parameters: “Tumor Treating Fields”, “TTFields”, “pulsed electric field”, “electroporation”, and “bioelectric signaling”; interleaved with (2) biological and immunological nodes: “receptor conformation”, “GPCR”, “tumor immune microenvironment”, “macrophage polarization”, “cGAS-STING”, and “immune checkpoint resistance”.

Inclusion and exclusion criteria were instituted to govern evidence selection. Inclusion criteria comprised: (1) peer-reviewed original research, clinical trials, case reports, and reviews; (2) studies characterizing subcellular mechanisms, organelle stress, immunomodulation, and adaptive resistance driven by electrical fields. Exclusion criteria consisted of: (1) literature lacking explicit electrical or biophysical parameters; (2) studies unrelated to oncological management or transmembrane bioelectric signaling. The literature screening and data extraction were cross-verified, thereby providing a reproducible methodological foundation for the precision electro-oncology paradigm.

Within the structural architecture of this synthesis, specific criteria were established to govern the prioritization of biological mechanisms discussed. The strategic prioritization of pathways such as membrane-bound allosteric sensing and cytoskeletal mechanotransduction over alternative phenomena was based on their support by a dual chain of scientific evidence. Specifically, these pathways are characterized by: (1) microscopic-level data, including full-atomistic molecular dynamics simulations that explore potential conformational displacements under physical field variations; and (2) macroscopic-level empirical evidence, such as multi-omics profiling and *in situ* observations demonstrating functional phenotypic remodeling within the local microenvironment.

Conversely, cellular responses that currently lack explicit force-transduction validation or remain primarily within the scope of phenomenological observation were classified as emerging evidence and positioned accordingly within the text. Furthermore, to mitigate individual selection bias during data extraction, the screening and cross-referencing of literature were executed independently by researchers with backgrounds in bioelectromagnetics and tumor immunology. Clarifying this prioritization logic establishes an objective methodological foundation for evaluating the precision electro-oncology paradigm.

## 2 Molecular antennas—capture of bioelectric signals by electro-sensitive receptors

Exploring how physical stress is precisely translated into intracellular biochemical signals is a central component in establishing the theoretical framework of Electro-Oncology. Specific structures on the cell surface and within the cell are considered to function as “molecular antennas,” responsible for capturing exogenous electric field signals and initiating subsequent cascade reactions.

### 2.1 Conformational sensing and signal activation of G-protein-coupled receptors

**Figure 1:**
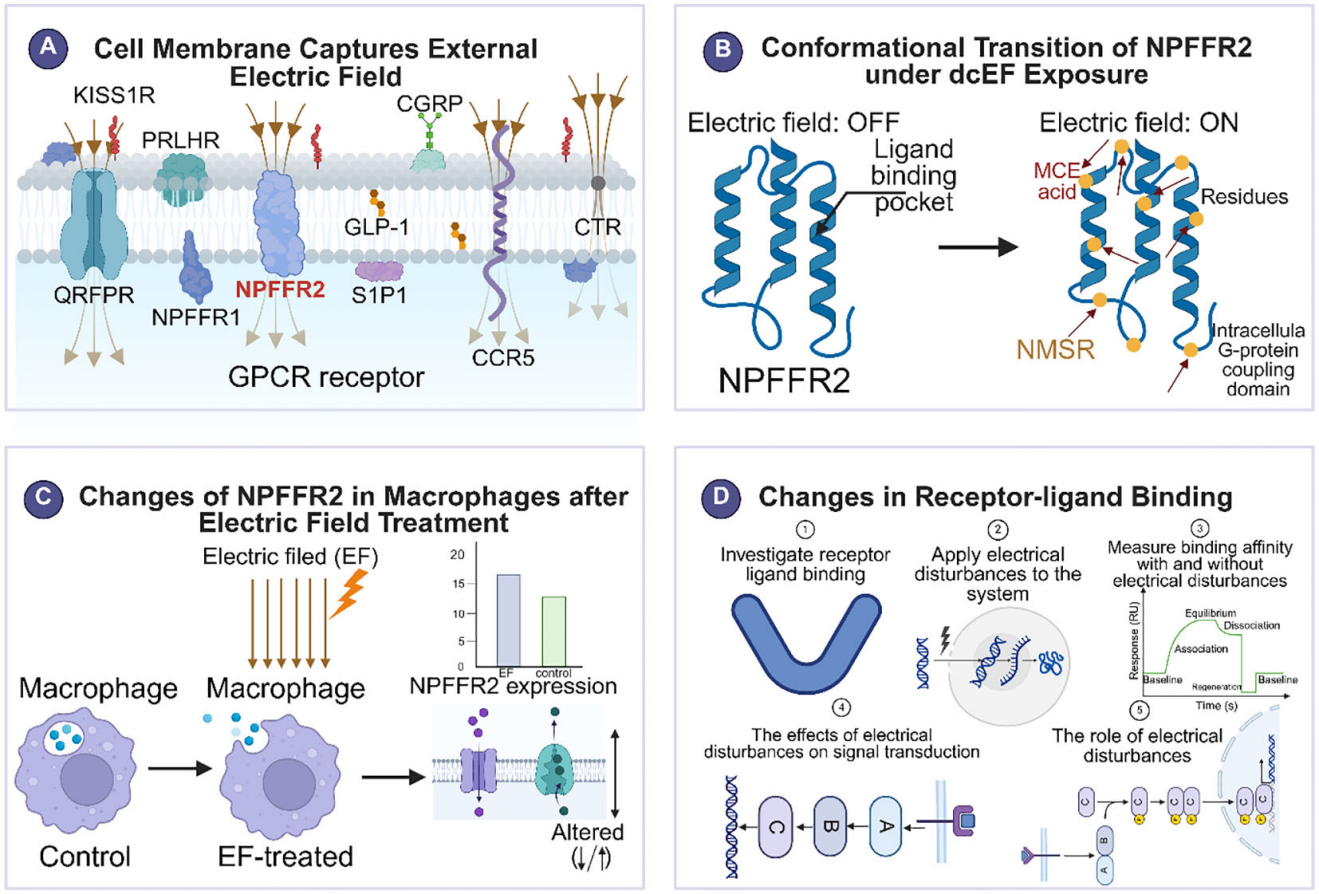
Molecular mechanisms of bioelectric signal perception via membrane receptors. **(A)** Schematic representation of the plasma membrane functioning as a “molecular antenna” to capture exogenous electric fields. **(B)** Molecular dynamics simulations illustrating the conformational transition of the G protein-coupled receptor NPFFR2 under direct current electric field (dcEF) exposure. **(C)** Quantitative and structural alterations in NPFFR2 expression and dynamics within macrophages following electric field intervention. **(D)** Differential analysis of receptor-ligand binding kinetics in the presence or absence of electrical perturbation.

As the primary barrier for sensing environmental changes, transmembrane proteins on the cell membrane occupy a critical position in electric field sensing (20) (**Figure 1A**). Previous investigations utilizing molecular dynamics simulations explored the potential regulatory responses of transmembrane domains under physical fields, suggesting that dcEF exposure correlated with a downregulation of target receptor expression in macrophages and potential variations in the predicted conformational kinetics of the receptor (5) (**Figure 1B**). More microscopic kinetic analyses suggest that electric fields may induce physical displacement of charged amino acid residues within the protein by altering the predicted conformation of the receptor protein, thereby achieving the initial transformation from physical stress to chemical signals (5) (**Figure 1C**; **Table 1**). Furthermore, the intervention of electric stimulation on transmembrane ion transport is also considered one of the biophysical foundations for reshaping cellular functions through non-pharmacological means (20) (**Figure 1D**).

**Table 1:** Molecular mechanisms of cellular sensing and transduction of electric field signals.

Biological target	Sensing mechanism	Signal transduction pathway	Key biological response	Model system classification	References
G-protein-coupled Receptors (GPCRs)	Conformational dynamics and allosteric changes	Suppression of receptor expression and altered transmembrane dynamics	Transformation of physical stress into biochemical cascades	*In vitro* mechanistic	(5, 20)
Cytoskeleton (Microtubules/Actin)	Physical disruption of tubulin and organization of F-actin	Activation of GEF-H1 and downstream NF-κB/AP-1 signaling	Mitotic interference and pro-inflammatory phenotype skewing	Murine model/*In vitro* mechanistic	(14, 21, 22)
Nuclear Envelope	Localized physical rupture and instability during S/G2 phase	Release of micronuclei and activation of cGAS-STING/AIM2	Initiation of innate immune sensing and interferon responses	*In vitro* mechanistic	(11, 14)
Cell Membrane Lipids	Increased permeability and lipid migration/rearrangement	Spatial clustering of surface receptors (PD-1, MHC II)	Enhanced formation of immune recognition synapses	*In vitro* mechanistic	(12, 15)
Electronic Orbitals	Modulation of Eg orbital occupancy in catalytic centers	Facilitation of valence electron transfer via electric fields	Optimization of ROS production and ER stress activation	*In vitro* mechanistic	(29, 30)

It is pertinent to note that although molecular dynamics simulations provide atomistic clues regarding the potential allosteric effects of receptors like NPFFR2 under directional field exposure (5), the current framework remains largely prediction-driven. A methodological gap exists between such theoretical modeling and experimentally verified electro-responsive membrane systems. Computational simulations often rely on simplified lipid environments and idealized force-field parameters, which may not fully account for the intrinsic heterogeneity of native plasma membranes or the dynamic interference of endogenous bioelectric gradients. Consequently, extrapolations based on in silico predictions require caution, and the calculated allosteric dynamics await systematic cross-validation through *in situ* biophysical assays (20).

### 2.2 Cytoskeleton as a relay hub for electro-mechanical signaling

**Figure 2:**
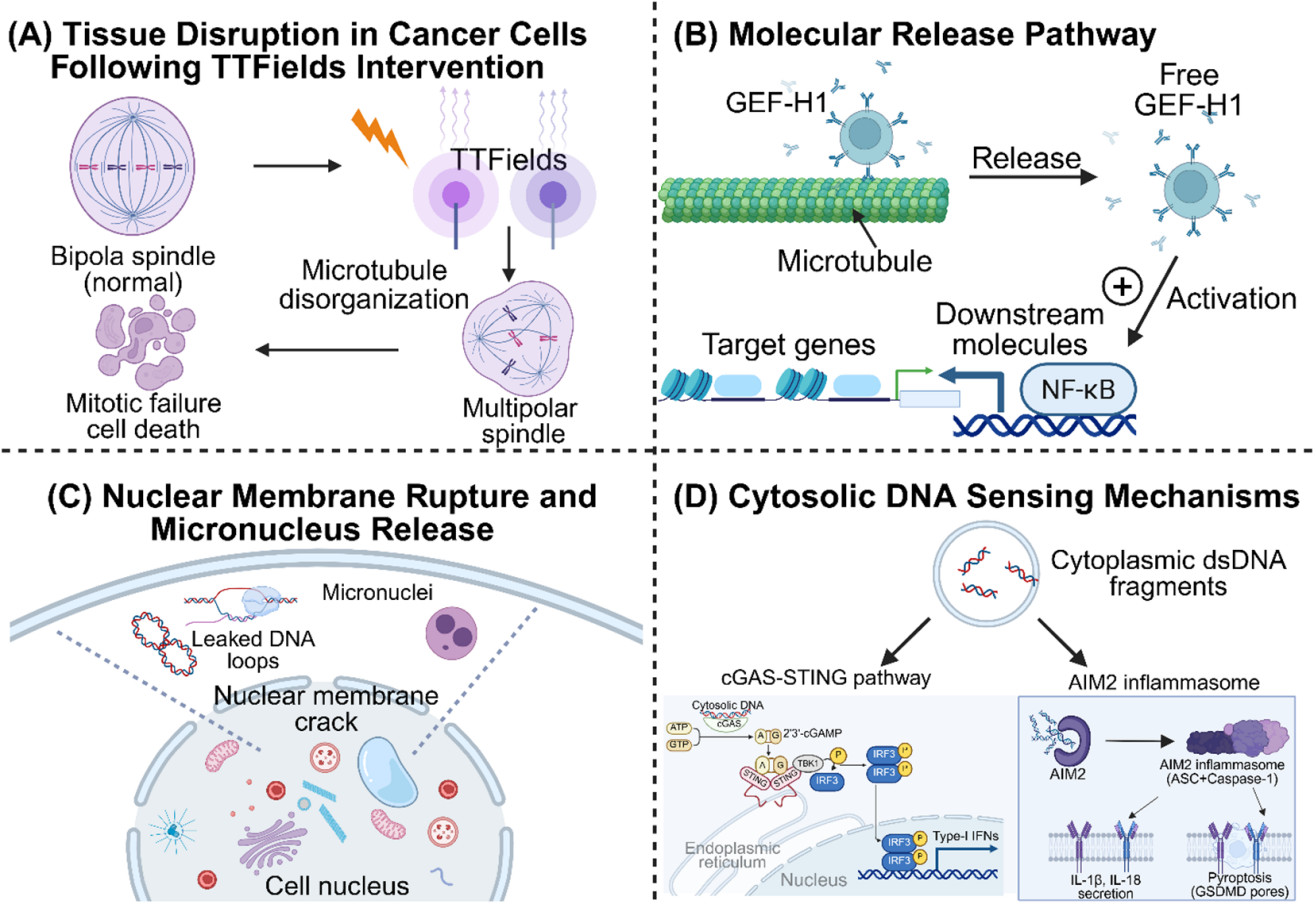
Cytoskeletal remodeling and nuclear integrity under electro-stress. **(A)** Disruption of microtubule organization and induction of multipolar spindle formation in mitotic cancer cells following TTFields intervention. **(B)** Mechanotransduction pathway highlighting the release and activation of microtubule-associated GEF-H1. **(C)** Localized physical rupture of the nuclear envelope and the subsequent release of micronuclei induced by physical compression during S/G2 phase. **(D)** Intracellular sensing mechanisms of cytosolic DNA fragments via the cGAS-STING and AIM2 inflammasome pathways.

The physical perturbation of the cytoskeleton by electric fields represents another primary axis of signal transduction. In models such as biliary tract cancer, Tumor Treating Fields (TTFields) have been observed to cause microtubule organization disruption and induce the formation of multipolar spindles, thereby interfering with cancer cell mitosis (21) (**Figure 2A**). Notably, this direct physical impact on the cytoskeleton further activates microtubule-associated guanine nucleotide exchange factor H1 (GEF-H1) (22) (**Figure 2B**). In macrophage models, the activation of GEF-H1 triggers downstream NF-κB and AP-1 signaling pathways, thereby driving the pro-inflammatory remodeling of the cellular phenotype (22) (**Figure 2B**; **Table 1**). Real-time live-cell imaging using high-throughput experimental platforms (such as the SCHEPHERD system) shows that electric fields can guide directional migration responses by reprogramming F-actin dynamics (23).

### 2.3 Rearrangement of membrane lipids and modification of glycosylation patterns

Changes in the membrane microenvironment induced by electro-stress have profound effects on receptor function. Sauer et al. explored the regulation of melanoma cells by nanosecond pulsed electric fields (nsPEF), observing that under non-cytotoxic parameters, electric fields can lead to increased membrane permeability and typical morphological changes characterized by lipid migration toward the periphery. This physical perturbation is accompanied by the spatial rearrangement and clustering of surface receptors such as PD-1 and MHC II, suggesting that electric fields may physically enhance the formation of immune recognition synapses (15) (**Table 1**). Multi-omics profiling further reveals that electric field intervention significantly regulates the N-glycosylation patterns of membrane glycoproteins (such as LAMP1 and LAMP2); such glycomic modifications may further influence cell adhesion and interactions between extracellular matrix (ECM) receptors (6). Changes in membrane integrity driven by physical parameters are also considered mechanisms for regulating caspase-dependent pathways (12).

Within the expanding paradigm of intracellular electro-oncology, nanosecond pulsed electric fields (nsPEFs) represent a key axis of development, largely defined by their capacity for organelle-selective disruption and precision waveform engineering. Unlike conventional electroporation modalities that predominantly target the plasma membrane, the ultra-short duration of high-voltage nanosecond pulses falls below the dielectric relaxation time of the outer membrane (24). This biophysical property allows the electric field to bypass outer capacitive barriers and directly polarize intracellular endomembranes, such as those of the endoplasmic reticulum (ER) and mitochondria (24). Recent mechanistic insights underscore that this targeted subcellular disruption precipitates severe ER stress, DNA damage, and accelerated mitochondrial calcium mobilization, thereby orchestrating a characterized cascade of electroporation-associated immunogenicity that contributes to anti-tumor immune activation (25, 26).

From a translational oncology perspective, modern waveform engineering has transitioned toward sophisticated bipolar pulse modulations and high-frequency nanosecond pulse burst compression (24, 27). This emerging direction not only addresses electroporation kinetics across sub-microscopic scales, but is also suggested to mitigate therapy-induced skeletal muscle contractions and localized thermal loads at the macro-tissue level (24). Consequently, this approach offers a selectable, immunogenic, and patient-compliant non-pharmacological strategy for combination oncotherapy within parameterized biophysical frameworks.

### 2.4 Nuclear envelope integrity impairment and physical activation of intracellular detection pathways

The capture of electric field effects is not limited to the cell surface but extends to the nuclear level. TTFields have been found to induce local physical rupture of the nuclear envelope, particularly leading to the release of micronuclei clusters into the cytoplasm in S/G2 phase cells (11) (**Figure 2C**). Furthermore, as observed across multiple tumor-bearing murine models, these endogenous DNA fragments exposed to the cytoplasm activate intracellular sensors cGAS and AIM2, thereby translating nuclear physical compression into an interferon gene response mediated by the cGAS-STING pathway (11) (**Figure 2D**; **Table 1**). The inductive activation of transcriptional regulators Stat1 and Irf1 by electric fields further explores potential paths for the conversion of physical signal capture into chemotactic gene expression (14).

This physical stress-induced innate immune response generally follows a specific temporal and causal sequence. During vulnerable phases of the cell cycle, mechanical forces exerted by electric field exposure can precipitate localized nuclear-envelope rupture. Subsequently, chromosomal fragments may leak into the cytoplasm, forming micronuclei architectures or free dsDNA fragments. This cytosolic DNA leakage event functions as an upstream signal, triggering the binding and catalytic activation of the cGAS sensor to synthesize cyclic GMP-AMP (cGAMP). Ultimately, cGAMP binds to the STING protein on the endoplasmic reticulum membrane, orchestrating its translocation to the Golgi apparatus and initiating the downstream TBK1-IRF3 signaling cascade. This progression translates the initial nuclear mechanical disruption into a transcriptional response involving Type I interferons and chemoattractants (28).

### 2.5 Electronic orbital occupancy and fine-tuning of the biocatalytic environment

Frontier research has also explored the possibility of using built-in polarized electric fields to regulate biological processes at a more microscopic electronic level. By developing dual-atom piezo-catalytic platforms, researchers have explored how to drive valence electron transfer via electric fields, thereby adjusting the electronic orbital occupancy of reaction center atoms, such as the Eg orbital (29). This electronic-level fine-tuning has been proven to significantly optimize the production efficiency of reactive oxygen species (ROS), thereby intensifying endoplasmic reticulum (ER) stress and activating downstream immune response pathways (29) (**Table 1**).

When evaluating multi-physical coupling platforms, such as ultrasound-driven piezoelectric systems, descriptive terms reminiscent of an “electro-stress storm” are occasionally invoked; however, their molecular and biophysical boundaries require strict academic clarification (30). This phenomenon is not an isolated micro-physical entity, but rather an integrated, cross-scale physical-to-biological transduction cascade initiated by localized electric field intensities.

To properly contextualize its scientific merit, it is instructive to distinguish this integrated network from established biological processes through a hierarchical framework. First, piezoelectric catalysis serves as the primary physical trigger, converting mechanical energy into local polarization potentials and modulating electronic orbital occupancy at catalytic centers (31). Second, this localized electrical potential impinges upon cellular structures, where its initial physical manifestation at the plasma membrane corresponds to electroporation-induced damage, characterized by alterations in membrane permeability (32).

Crucially, this physical disruption propagates downstream to evoke organelle-scale biochemical stress responses, specifically manifesting as oxidative stress amplification and endoplasmic reticulum (ER)-stress signaling (24). The polarization kinetics perturb electron transport chains within the mitochondria and the ER, driving the generation of endogenous reactive oxygen species (ROS) alongside the engagement of the unfolded protein response. Ultimately, the convergence of these sequential mechanisms results in ROS-driven immunogenicity, facilitating the presentation of signals that promote immune cell recruitment (30). This clarified concept strictly contextualizes the multi-scale loop—originating from localized physical polarization and terminating in systemic immunogenic output—providing an objective mechanistic foundation for future evaluations in electro-oncology.

### 2.6 Methodological strategies to address prediction-driven limitations

To further evaluate the hypothesis of electro-sensitive GPCRs and address the divide between computational prediction and empirical evidence, the integration of high-resolution, real-time biophysical techniques provides a potential future direction. First, voltage-clamp coupled fluorescence resonance energy transfer (FRET) analysis serves as a prospective approach for tracking membrane protein conformational dynamics (33); by introducing site-specific fluorophores, this approach aims to allow the simultaneous application of electrical commands and real-time recording of nanoscale domain movements. Second, cryo-EM under controlled electric perturbation represents an emerging technological frontier (34); utilizing specialized grid freezing devices equipped with microelectrodes may potentially vitrify receptor proteins during electrical pulse delivery, thereby capturing transient intermediate conformations at near-atomic resolution. Lastly, single-molecule receptor tracking offers a direct means to examine the spatial reorganization of membrane proteins, allowing for the quantification of diffusion coefficients and electric field-induced clustering kinetics (35). The future convergence of these multi-scale validation methodologies is anticipated to provide a more rigorous empirical foundation for clarifying how physical stress translates into biochemical signals.

## 3 Physical programming—regulation of cell fate based on electric field parameters

**Figure 3:**
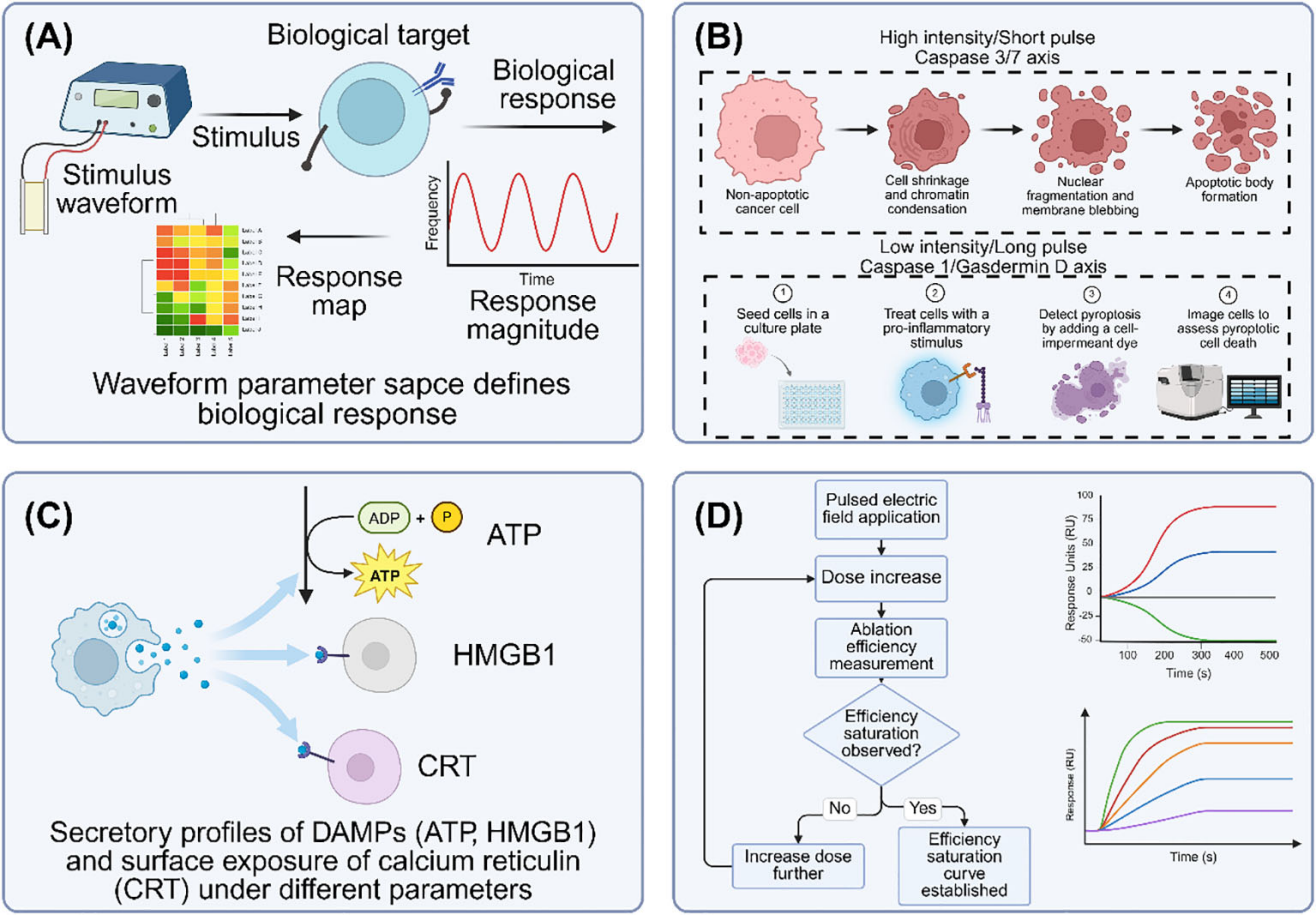
Parametric programming of immunogenic cell death pathways. **(A)** Mapping the biological response landscape within the waveform parameter space (frequency, intensity, and pulse width). **(B)** Differential induction of cell death modes: high-intensity short pulses activate the Caspase 3/7 axis (non-inflammatory apoptosis), while low-intensity long pulses promote Caspase 1/Gasdermin D activation (pro-inflammatory pyroptosis). **(C)** Secretory profiles of damage-associated molecular patterns (DAMPs, such as ATP and HMGB1) and surface exposure of calreticulin (CRT) across varied parameters. **(D)** Dose-response kinetics and saturation curves for pulsed electric field (PEF) ablation efficiency.

The biological effects of electric field therapy are not merely a result of singular physical interference but represent a highly sophisticated process dependent on specific combinations of physical parameters. Exploring how parameters such as frequency, intensity, pulse width, and dosage function as a “physical code” to regulate cell fate is pivotal for the realization of precision electro-oncology (**Figure 3A**).

### 3.1 Synergistic optimization of frequency and intensity: cell-specific sensitivity windows

Different cell types exhibit significant heterogeneity in their sensitivity to electric field frequencies. In studies targeting biliary tract cancer (BTC) cells, frequency scanning experiments exploring the 100–200 kHz range found that 150 kHz induced the most significant cytotoxicity, inhibition of colony formation, and microtubule disruption (21) (**Table 2A**). Conversely, glioblastoma (GBM) U87 cells demonstrated optimal proliferation inhibition at 200 kHz, with this suppressive effect showing clear time-dependence (3, 6) (**Table 2A**).

**Table 2A:** Parametric programming of cell fate and biological efficacy.

Parameter type	Specific values/ranges	Targeted cell type/model	Biological effect	References
Frequency	150 kHz	Biliary Tract Cancer (BTC)	Optimal cytotoxicity and microtubule disruption	(1, 21)
Frequency	200 kHz	Glioblastoma (GBM)	Maximum inhibition of proliferation	(3, 6)
Field Intensity	1.0–3.0 V/cm	Various Tumors/Migration	Dose-dependent induction of ICD and directional migration	(21, 23)
Pulse Width	Short Pulse + High Intensity	General Tumor Models	Induction of non-inflammatory apoptosis via Caspase 3/7	(12, 20)
Pulse Width	Long Pulse + Low Intensity	General Tumor Models	Induction of pro-inflammatory pyroptosis via Caspase 1	(12)
Pulse Pack Dose	60–100 Pulses	Breast Cancer/Melanoma	Saturation of ablation diameter and induction of abscopal response	(16, 44)

**Table 2B:** Cross-scale mechanistic comparison of distinct oncological electrical modalities.

Electrical modality	Penetration depth	Membrane interaction pattern	Subcellular stress signatures	Immune engagement profile	Reference
Tumor Treating Fields (TTFields)	Substantial	Biophysical interactions primarily targeting dividing cells	Disruption of mitosis	Localized immune engagement	(49)
Irreversible Electroporation (IRE)	Physically restricted by electrode geometry	Irreversible hydrophilic pore formation	Physical cell lysis	Localized inflammation and macrophage infiltration	(50)
Reversible Electroporation (RE)	Physically restricted by electrode geometry	Transient, self-sealing pore formation	Mild intracellular stress	Minimal direct activation (primarily facilitates macromolecular drug delivery)	(51)
Nanosecond Pulsed Electric Fields (nsPEFs)	Subcellular targeting capabilities	Acts directly on endomembrane systems (bypasses plasma membrane)	Mitochondrial depolarization and endoplasmic reticulum (ER) stress	Potential systemic anti-tumor immunity through pathways such as pyroptosis	(24)
Conventional AC/DC Stimulation	Modest (surface/tissue level)	Modulation of transmembrane channels and surface charges	Minimal intracellular stress	Phenotypic tuning (e.g., macrophage polarization)	(47)
Piezoelectric Field Systems	Deep (partially circumvents external electrode constraints)	Localized dynamic polarization	Generation of reactive oxygen species (ROS) and ER stress	Subsequent innate immune responses	(52)

Field intensity parameters directly determine the depth of the subsequent biological response. Research investigating the effects of 1.3–2.1 V/cm on BTC cells confirmed the intensity-dependent nature of the therapeutic efficacy (21). *In vivo* models suggest that 1.5 V/cm (RMS) may serve as a bioeffective threshold for inducing immunogenic cell death (ICD) and T cell infiltration (13, 23) (**Table 2A**). Clinical trials, such as LUNAR, have further established 150 kHz and a daily treatment duration of ≥18 h as the parametric benchmarks for lung cancer intervention (1, 36).

### 3.2 Pulse parameter programming: biophysical thresholds involving membrane destabilization, ionic flux, and death execution pathways

The influence of exogenous electric field parameters on cancer cell death phenotypes is not a simple linear process, but rather a non-linear biochemical cascade constrained by specific biophysical thresholds. The underlying rationale is that different forms of electrodynamic energy can selectively affect the critical thresholds of the plasma membrane or organelle membranes, thereby inducing divergent intracellular stress responses.

When cells are exposed to short pulses combined with high field intensities (e.g., nanosecond pulsed fields), the initial biophysical response occurs primarily at the ultrastructural level. The electrical stress generated by such waveforms can exceed the dielectric threshold of internal membrane systems, creating a high density of transient micro-pores across membrane matrices (37). This physical permeabilization leads to the rapid alteration of transmembrane ionic gradients, accompanied by the transient depolarization of the mitochondrial membrane potential and an increase in reactive oxygen species (ROS). The subsequent change in mitochondrial outer membrane permeability facilitates the release of cytochrome c into the cytosol, which may promote apoptosome assembly and activate the Caspase 3/7 signaling axis (**Figure 3B**), guiding the cell toward a morphologically silent and less inflammatory apoptotic trajectory (38, 39).

Conversely, shifting the exposure parameters to longer pulses paired with lower field intensities (e.g., microsecond or millisecond pulses) often evokes different non-linear kinetic characteristics. This sustained electro-stress tends to induce prolonged plasma membrane destabilization and severe ionic flux imbalance. Notably, the rapid influx of extracellular calcium (Ca^2+^) through the compromised membrane architecture can trigger significant intracellular calcium overload (40, 41). This persistent physical stress and ionic perturbation are detected by cytosolic innate immune sensors, serving as a contributing stimulus for the priming and assembly of NLRP3 inflammasomes. Inflammasome maturation mediates the cleavage of pro-Caspase 1, which subsequently targets Gasdermin D (GSDMD). The resulting GSDMD N-terminal fragments oligomerize to form pores on the plasma membrane, allowing the release of interleukin-1β (IL-1β) and related danger signals (such as HMGB1), suggesting the activation of the pro-inflammatory pyroptotic pathway (42, 43). Investigating these biophysical thresholds—encompassing transmembrane potential, mitochondrial permeability, and enzymatic cleavage—provides further insight into how electric field parameters may function as physical switches in modulating tumor cell death phenotypes.

### 3.3 Dose-response dynamics: establishing the minimum effective physical load

A complex nonlinear relationship appears to exist between electric field dosage (e.g., the number of pulse packets) and the biological response. In pulsed electric field (PEF) ablation studies, a comparison of 20–100 pulse packets revealed that 60 packets reached a saturation point for ablation diameter; further increasing the dose only significantly increased the tissue thermal load without markedly expanding the effective ablation volume (44). This dosage “ceiling” effect suggests that researchers should focus on the “minimum effective dose” to balance efficacy and safety (45).

This dosage regulation is also reflected in systemic immune activation. A single ablation (e.g., 100 pulse packets) combined with repeat activation can trigger significant abscopal effects in patients with refractory melanoma, leading to immune remission in distant, non-directly treated lymph nodes (16) (**Table 2A**). Research on the interaction between dosage and immune memory formation suggests that electrical stimulation (ES) may drive macrophage polarization toward the pro-regenerative M2 phenotype within specific parametric windows via the JAK/PI3K/AKT axis, demonstrating the potential bidirectionality of immune modulation (46, 47).

### 3.4 Spatial flow and dynamic remodeling: programming collective cell behavior

Advanced high-throughput electrobiological platforms (e.g., SCHEPHERD) have explored the directional control of collective cell behavior through electric field parameters. Through precise scanning within intensity gradients of 0.25–3 V/cm, researchers have established the “throttle and steering wheel” effect of dcEF on cell migration direction and velocity, revealing the potential of electric fields as physical instructions to guide tissue-scale collective movement (23).

This dynamic remodeling is not limited to motility. At specific cell cycle phases (e.g., S/G2 phase), electric fields can induce local instability and rupture of the nuclear envelope, triggering DNA sensing pathways (11). This “programming” of physical properties may render tumor cells with a “conditional vulnerability,” providing a critical molecular window for subsequent combination therapies involving immune checkpoint inhibitors or PARP inhibitors (2, 6). The synergy between physical topological parameters (e.g., graphene microgrooves) and electric field intensity further expands the parametric space for the refined regulation of macrophage phenotypes (5, 20, 48).

### 3.5 Cross-scale mechanistic comparison of distinct oncological electrical modalities

To establish a comprehensive biophysical perspective, it is instructive to examine the differences among various oncological electrical intervention modalities. Due to fundamental variations in frequency, pulse width, field strength, and polarization characteristics, these modalities exhibit distinct mechanistic pathways and stress signatures at both cellular and tissue scales.

Tumor Treating Fields (TTFields) involve intermediate-frequency (100–300 kHz), low-intensity (1–3 V/cm) alternating electric fields, which generally offer substantial tissue penetration depth. They primarily disrupt mitosis through biophysical mechanisms, inducing cell death accompanied by localized immune engagement (49). In contrast, irreversible electroporation (IRE) and reversible electroporation (RE) typically utilize high-voltage microsecond pulses, with penetration depths physically restricted by electrode geometry. IRE creates irreversible hydrophilic pores in the plasma membrane, driving physical lysis that triggers localized inflammation and macrophage infiltration (50). Conversely, membrane pores induced by RE are transient and self-sealing, resulting in mild intracellular stress, making the technique suitable for facilitating macromolecular drug delivery (51).

Within the ultra-short pulse regime, nanosecond pulsed electric fields (nsPEFs) demonstrate subcellular targeting capabilities driven by their nanosecond duration and high peak intensities. Because the pulse width is shorter than the dielectric relaxation time of the plasma membrane, nsPEFs can act directly on endomembrane systems. This interaction induces mitochondrial depolarization and endoplasmic reticulum (ER) stress, potentially triggering systemic anti-tumor immunity through pathways such as pyroptosis (24). Conventional low-frequency alternating or direct current stimulation focuses on modulating transmembrane channels and surface charges using weak currents; this yields minimal intracellular stress and is primarily investigated for phenotypic tuning, such as macrophage polarization (47).

Furthermore, ultrasound-activated piezoelectric field systems represent an emerging modality. By generating localized polarization fields via piezoelectric materials, these systems partially circumvent the penetration constraints of external electrodes. Their mechanism involves the generation of reactive oxygen species (ROS) through localized dynamic polarization, suggesting a potential pathway for inducing ER stress and subsequent immune responses (52). This cross-scale mechanistic divergence, summarized in **Table 2B**, provides a methodological foundation for exploring combination therapeutic strategies tailored to tumor physical heterogeneity.

## 4 Systemic remodeling—evolution of the tumor immune microenvironment from “cold” to “hot”

The homeostatic imbalance of the tumor immune microenvironment (TIME) represents a core obstacle to the efficacy of immunotherapy, particularly in “cold” tumors characterized by immune exclusion or desertion. Exploring how physical electric field modalities can be utilized to remodel this microenvironment and drive its evolution toward a “hot” tumor phenotype carries profound clinical significance for improving the response rates of systemic immunotherapies.

### 4.1 Immunogenic cell death and antigen release mediated by physical stress

Electric field intervention, serving as a potent physical stressor, can initiate immunogenic cell death (ICD) through non-thermal ablation pathways. Numerous experimental observations have indicated that under the influence of Tumor Treating Fields (TTFields) or Pulsed Electric Fields (PEF), various tumor cell types exhibit the characteristic release of damage-associated molecular patterns (DAMPs) (3, 21) (**Figures 3C, D**). This physically induced death pathway involves the surface exposure of calreticulin (CRT) and the significant extracellular secretion of high mobility group box 1 (HMGB1) and adenosine triphosphate (ATP) (21). The synergistic release of these DAMPs provides essential “eat-me” and “find-me” signals for dendritic cells (DCs), thereby initiating antigen capture processes within cold tumors (3) (**Table 3A**). Furthermore, the precise induction of pyroptotic modes through regulated pulse waveforms may further intensify the release of pro-inflammatory cytokines, establishing a biochemical foundation for microenvironmental remodeling (12).

**Table 3A:** Multi-dimensional remodeling of the tumor immune microenvironment (TIME).

Remodeling domain	Specific changes	Molecular markers/factors	Impact on immune response	References
Immunogenic Cell Death (ICD)	Non-thermal release of DAMPs	CRT exposure; HMGB1 and ATP secretion	Facilitation of antigen capture by Dendritic Cells (DCs)	(3, 21)
Immune Infiltration	Recruitment of effector T cells	Upregulation of CCL2/8 and CXCL9/10	Transformation of “cold” tumors into “hot” tumors	(13, 14)
Innate Cell Skewing	Reprogramming of macrophage polarization	GEF-H1 activation; MHC II and CD80 expression	Conversion of M2 (suppressive) to M1 (pro-inflammatory) phenotype	(22, 47)
Tertiary Lymphoid Structures	Formation of local immune niches	Mature TLS formation in TIME	Enhanced T cell expansion and “immune factory” effect	(16)
Systemic Immunity	Induction of Abscopal Response	TAA-specific IgG and Tem cell expansion	Long-term immune memory and prevention of recurrence	(16, 45)

**Table 3B:** Quantitative bioelectric parameters for guiding distinct macrophage functional states.

Macrophage functional state	Wave modality/stimulation type	Frequency	Localized field intensity/current	Pulse duration & cumulative exposure	Reference
Proinflammatory	Alternating Electric Fields/Piezoelectric Stimulation	Intermediate (e.g., 100–300 kHz)	90 W	Continuous (e.g., 24–72 h) or intermittent daily sessions	(55)
Interferon-responsive	Alternating Electric Fields/Piezoelectric Stimulation	Intermediate to High	1–2 V/cm	Intermittent bursts or continuous exposure	(22, 55)
Pro-Regenerative M2 Phenotype	Direct current (DC) intervention	Direct current (DC)	100 mV/mm	Continuous, time-dependent exposure	(47)
Inflammatory	Nanosecond pulsed electric field	1–100 Hz	0.9 MV/m	200–1000 ns	(59)
Anti-inflammatory	Extremely low frequency pulsed electromagnet fields	52 Hz	pulse burst pattern	30 min	(60)

### 4.2 Activation of chemokine axes: recruiting immune “reinforcements”

**Figure 4:**
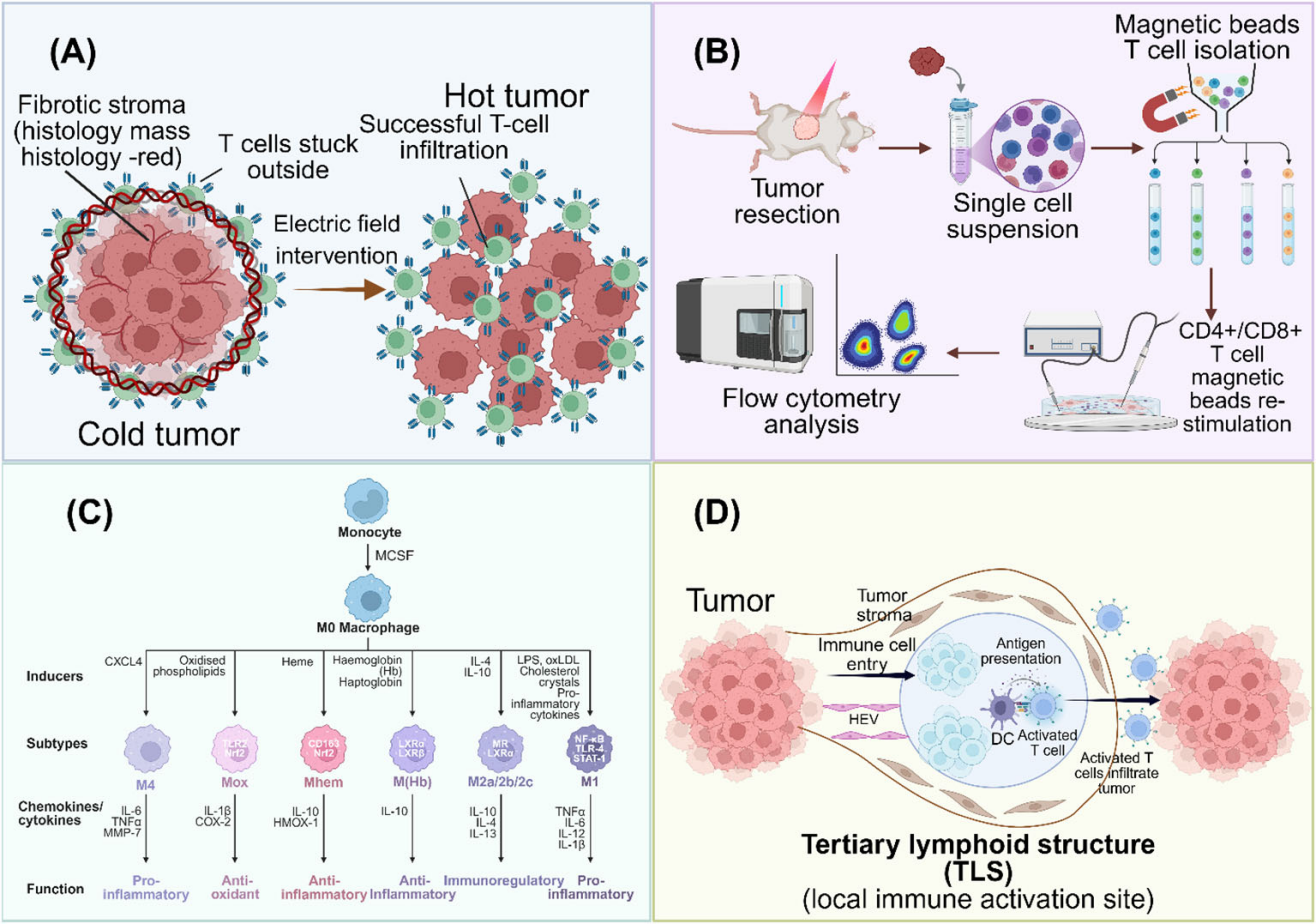
Systemic remodeling of the tumor immune microenvironment. **(A)** Phenotypic transition of tumors from a “cold” (immune-excluded) to a “hot” (immune-infiltrated) state following electric field intervention. **(B)** Synergistic enhancement of tumor-infiltrating T cell effector functions through combined TTFields and ICI therapy. **(C)** Tumor-associated macrophages (TAMs) exist in the tumor microenvironment as a highly dynamic, multistate, continuous spectrum. **(D)** Establishment of tertiary lymphoid structures (TLS) within the tumor stroma as localized hubs for immune activation.

To achieve a “cold-to-hot” transition of the microenvironment, the spatial recruitment of immune cells is an indispensable link (**Figure 4A**). Research has explored how electric fields induce chemokine expression by activating intracellular signal transduction pathways. In non-small cell lung cancer (NSCLC) models, ICD induced by TTFields activates the Stat1 and Irf1 transcriptional regulators, leading to a significant upregulation of critical chemokines such as CCL2/8 and CXCL9/10 (14) (**Table 3A**). The activation of these chemotactic axes suggests the possibility of physically driving changes in the spatial distribution of immune cells, effectively guiding the infiltration of CD4+ and CD8+ T cells into the tumor core (13, 14) (**Figure 4B**). Clinical biopsy evidence has also demonstrated the formation of mature tertiary lymphoid structures (TLS) following PEF ablation, hinting that electric fields may establish local “immune factories” that support T cell expansion and activation (16) (**Figure 4D**; **Table 3A**).

It is pertinent to note that the activation of the cGAS-STING pathway within the electro-oncology framework does not invariably yield an exclusively positive immunological response. Recent tumor immunology investigations indicate a biological duality of STING signaling across both temporal and spatial dimensions (53). Although acute electrical exposure may modulate tumor phenotypes favorably through this axis, persistent and unresolved electro-stress exposure may trigger counter-regulatory, compensatory suppressive mechanisms. Such sustained inflammatory microenvironments can induce the upregulation of inhibitory receptors on local T cells, accelerating their transition toward immune exhaustion. Furthermore, persistent STING hyperactivation has been associated with the recruitment of myeloid-derived suppressor cells (MDSCs) and the infiltration of regulatory T cells (Tregs), which may reconstruct a chronic inflammatory immunosuppressive barrier (53). Consequently, the modulation of electrical pulse intervals to prevent persistent STING overstimulation represents a factor that requires careful consideration in future evaluations of biophysical immunomodulation.

### 4.3 Parameterized remodeling of multi-state macrophage spectra under electric fields

Within the context of precision electro-oncology, the modulation of innate immune components by physical electro-stress extends beyond the conventional M1/M2 binary polarization model (**Figure 4C**). Driven by advancements in single-cell transcriptomics and spatial multi-omics, contemporary consensus indicates that tumor-associated macrophages (TAMs) exist as a highly dynamic, multi-state continuous spectrum within the tumor microenvironment (54). Exogenous electric field interventions can sculpt these heterogeneous macrophage subpopulations through distinct biophysical transduction circuits.

First, electrical exposure has been suggested to modulate the phenotypic characteristics of inflammatory TAMs. This regulation may involve physical perturbations of cytoskeletal networks and ion channels, subsequently influencing the expression of classical pro-inflammatory cytokines such as TNF-α and IL-6 (22, 55). Second, associated with electro-stress-induced cytosolic DNA leakage, electrical intervention may facilitate the local enrichment of interferon-responsive TAMs. This population is characterized by cGAS-STING pathway activation and the expression of interferon-stimulated genes (e.g., CXCL9/10), providing potential spatial cues for effector T cell recruitment (56).

Regarding angiogenic TAMs and tissue-remodeling TAMs, which typically sustain the immunosuppressive niche, parameterized electric fields have shown the potential to downregulate their pro-angiogenic and matrix-modifying secretory profiles, thereby influencing the physical shielding of solid tumors (57). Furthermore, physical stress may induce the emergence of metabolically reprogrammed TAMs. By fine-tuning plasma membrane potentials and transmembrane ionic flux, electro-stress can alter the efficiency of macrophage substrate utilization (e.g., lactate), potentially steering them toward immune-supportive phenotypes (58). Investigating the regulation of this continuous macrophage spectrum provides a more objective theoretical foundation for assessing the role of physical fields in microenvironmental remodeling.

To provide a systematic and quantitative reference of physical parameters for experimental investigators, this review introduces a dedicated bioelectric parameter directory, designated as **Table 3B**. Based on recent primary investigations, this matrix outlines the specific biophysical boundary conditions required to guide tumor-associated macrophages (TAMs) into distinct functional subclusters, including inflammatory, interferon-responsive, angiogenic, tissue-remodeling, and metabolically reprogrammed states.

Specifically, the table delineates core engineering parameters such as wave modality, localized field intensity, frequency, pulse duration, and cumulative exposure cycles. For instance, it summarizes the parameter signatures of alternating electric fields or ultrasound-driven piezoelectric stimulation in inducing pro-inflammatory phenotypes (22, 55), as well as the intensity and time-dependent nature of direct current interventions in driving macrophages toward tissue-repair and metabolically reprogrammed states (47). By compiling these quantitative engineering parameters, this section aims to offer an objective and standardized cross-scale reference for future combination experimental designs and dosimetric optimization in electro-immunology.

### 4.4 Clinical realities of the abscopal effect: discrepancies in frequency between monotherapy and combination paradigms

While conceptualizing localized physical field intervention as an “*in situ* vaccine” offers mechanistic inspiration, transitioning this paradigm to clinical application necessitates a cautious evaluation of its true documented frequency across distinct treatment architectures. Current evidence suggests that the incidence of the abscopal effect exhibits a pronounced divergence when comparing solitary physical interventions with integrated immunophysical combination regimens.

Within the framework of single-agent electric field therapy (such as isolated alternating field exposure or solitary ablation), the spontaneous occurrence of an abscopal effect remains clinically rare (61). This monotherapeutic limitation is partly attributed to the multi-layered immunosuppressive barriers intrinsic to the tumor microenvironment. Although localized physical fields trigger cell lysis and the transient emission of damage-associated molecular patterns (DAMPs) and tumor-associated antigens (TAAs), these signals are frequently insufficient to independently overcome established peripheral self-tolerance. In the absence of sustained systemic immune support, surviving peritumoral fractions and associated suppressive networks may neutralize the liberated antigenic load, limiting the potential for systemic regression of distant lesions (62).

Conversely, when electrical modalities are incorporated as components of multi-modal combination architectures alongside systemic immune checkpoint inhibitors (ICIs), the documented frequency of the abscopal effect and associated distant response rates show improvement (19, 63). Recent translational studies and *in vivo* modeling indicate that the physically induced antigenic burst can establish a synergy with systemic anti-PD-1 or anti-PD-L1 therapeutics. The bioelectric field facilitates localized antigen exposure, while pharmacological inhibitors assist in reversing effector T-cell exhaustion. This combined approach is suggested to support the expansion of circulating TAA-specific effector memory T-cell (Tem) clones, thereby potentially elevating the probability of immune-mediated regression in distant metastases (19, 64). Recognizing this divergence in occurrence frequency underscores the importance of positioning parameterized physical engineering within comprehensive, multi-modal immunotherapeutic strategies.

### 4.5 Clinical evidence: physical-biological synergy initiating the “hot” tumor paradigm

Clinical trial data (e.g., the LUNAR and METIS studies) further explore the immense potential of the synergistic interplay between electric fields and immune checkpoint inhibitors (ICIs). In NSCLC patients with brain metastases suitable for stereotactic radiosurgery, the addition of TTFields not only significantly delayed intracranial progression but also showed more pronounced benefits in the subgroup receiving ICI therapy (2). This suggests that physical fields may enhance the sensitivity of drug-resistant patients to biological immunotherapies by increasing the expression of receptors such as PD-1 and improving microenvironmental infiltration (1, 15).

Although parameterized immune remodeling presents a characterized biological framework regarding the expansion of systemic effector memory T cells (Tem) and innate signaling modulation, evaluating its potential for the clinical management of refractory malignancies requires an objective perspective grounded in current clinical trial outcomes.

Evidence from ongoing clinical evaluations indicates that the real-world efficacy of combining electrical modalities with systemic immunotherapies is characterized by heterogeneity among patient cohorts. In multi-center investigations targeting recurrent glioblastoma or advanced non-small cell lung cancer, while the integration of localized fields correlates with preliminary survival benefits and tolerability in select patient strata (2), consistent improvements in overall objective response rates (ORRs) and progression-free survival (PFS) have not been uniformly observed across broad intention-to-treat (ITT) populations (36). This discrepancy suggests that physical-induced antigen liberation and immune sensitization are influenced by complex microenvironmental cross-currents in human patients.

Consequently, maintaining a measured academic perspective in translational research is essential. Future efforts may need to pivot toward identifying specific predictive biomarkers through multi-omics profiling rather than assuming uniform efficacy. Building on this, refining the spatio-temporal dosimetric configurations between physical parameters and immune checkpoint inhibitors represents a practical approach to systematically exploring therapeutic windows for recalcitrant tumors.

### 4.6 Long-term biological adverse effects and counter-regulatory barriers of induced immune remodeling

While elucidating the therapeutic potential of electric fields in microenvironmental remodeling, maintaining a rigorous clinical perspective necessitates an objective evaluation of the potential long-term biological adverse effects precipitated by sustained immune modulation. When the temporal duration and localized physical load of these interventions cross specific homeostatic thresholds, they may elicit counter-regulatory localized or systemic toxicities (65).

A primary consideration involves localized autoimmunity and chronic barrier inflammation (66). Parameterized electric fields drive immunogenic cell death (ICD), precipitating the continuous emission of damage-associated molecular patterns (DAMPs). Although acute DAMP accumulation is necessary to initiate antigen presentation, prolonged stagnation over extended clinical courses can influence peripheral self-tolerance boundaries. This chronic danger signaling may activate resident immune cells within adjacent healthy tissues, potentially triggering autoimmune inflammation or fibrotic rewiring that compromises long-term tissue integrity (65).

Furthermore, chronic bioelectric intervention carries the risk of inducing compensatory systemic immune exhaustion. Persistent subcellular stress and the associated leakage of cellular contents can maintain downstream transduction nodes (such as the cGAS-STING pathway) in a state of prolonged activation (59). Over extended periods, this non-homeostatic signaling frequently engages negative feedback loops, accelerating the upregulation of inhibitory receptors on CD8+ T cells and driving them toward functional exhaustion. Additionally, unresolved physical stress has been suggested to facilitate the recruitment of myeloid-derived suppressor cells (MDSCs), potentially constructing an alternate immunosuppressive barrier across non-targeted regions (67).

Consequently, to ensure clinical safety, therapeutic protocols must establish dynamic dosimetric safety boundaries. Future human clinical studies should consider the longitudinal monitoring of peripheral effector T-cell distributions, regulatory T-cell heterogeneity, and systemic serum profiles of pro-inflammatory cytokines. Incorporating these immunological kinetics into real-time monitoring systems will assist in precisely modulating physical pulse intervals, thereby establishing a rational therapeutic window that balances anti-tumor efficacy with the mitigation of long-term systemic toxicities (68).

## 5 Resistance evolution—precision combination strategies from a multi-omics perspective

Exploring the biological evolutionary patterns of tumor cells under long-term exposure to electric fields is of paramount importance for overcoming potential therapeutic resistance and designing refined combination treatment regimens. With the advancement of multi-omics technologies, researchers have begun to decode electric field-induced cellular stress defense mechanisms across multiple dimensions, including the proteome, phosphoproteome, and glycome (6, 20, 69).

Within the framework of precision electro-oncology, a deconstruction of the evolutionary dynamics governing tumor adaptation under continuous electrical exposure provides theoretical value for designing combination regimens. Literature suggests that the upregulation of PARP1, BRD4, and PD-L1 does not follow a uniform spatio-temporal trajectory; rather, these responses represent distinct stages of acute cellular stress, epigenetic reorganization, and microenvironmental compensation.

### 5.1 Compensatory defense mechanisms induced by electric fields: insights from multi-omics profiling

**Figure 5:**
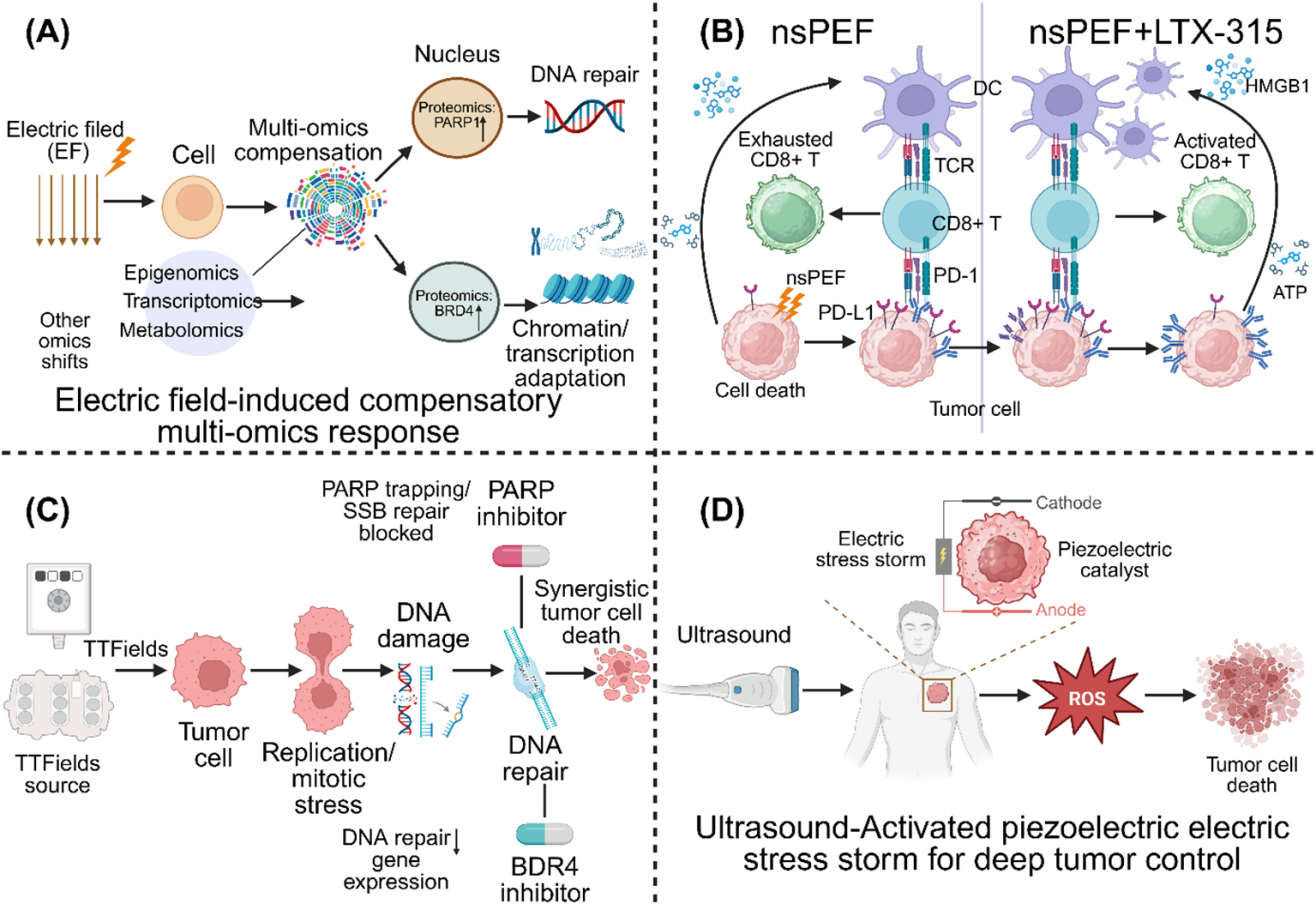
Adaptive evolution and precision combination strategies. **(A)** Multi-omics landscape of electric field-induced compensatory responses, highlighting the proteomic upregulation of PARP1 and BRD4. **(B)** Feedback upregulation of PD-L1 following PEF ablation, contributing to transient immune evasion. **(C)** Synergistic potentiation of tumor cell death via combined TTFields and PARP or BRD4 inhibition. **(D)** Implementation of an ultrasound-activated piezoelectric “electro-stress storm” for deep-seated tumor control and reactive oxygen species (ROS) generation.

Initially, upon early-stage electric field intervention, the elevation of poly (ADP-ribose) polymerase 1 (PARP1) levels functions primarily as an acute transient stress response (17). This upregulation is precipitated by acute DNA lesions and replication stress, serving as a countermeasure to preserve basal genomic integrity. However, under chronic and sustained electrical exposure, this temporary survival feedback may transition into stable epigenetic rewiring, orchestrated by factors such as bromodomain-containing protein 4 (BRD4) (70). The recruitment of BRD4 to specific stress-responsive loci may lock the temporary survival signal into a sustained resistant phenotype at the chromatin level (71) (**Figure 5A**; **Table 4**). Furthermore, phosphoproteomics has explored changes in the activity of key kinases such as ABL1 and PDK1, while glycomics has revealed disruptions in the N-glycosylation patterns of membrane proteins such as LAMP1 and LAMP2. These molecular signatures may collectively constitute the complex adaptive response of tumor cells to the electric field environment (6).

**Table 4:** Compensatory resistance mechanisms and precision synergistic strategies.

Resistance node	Evolutionary mechanism	Precision synergistic strategy	Therapeutic outcome	References
DNA Damage Repair	Compensatory upregulation of PARP1	TTFields + PARP Inhibitors (e.g., Olaparib)	Enhanced cell death via “conditional vulnerability”	(6, 77)
DNA Damage Repair	Overexpression of BRD4	TTFields + BRD4 Inhibitors (e.g., JQ-1)	Inhibit the reproduction and growth of cancer cells	(6)
Immune Evasion	Feedback upregulation of PD-L1	PEF + ICI or Oncolytic Peptides (LTX-315)	Restoration of T cell cytotoxicity and systemic relief	(3, 73)
Drug Resistance (Clinical)	Progression on ICI/Chemotherapy	TTFields + ICI (LUNAR/METIS Trials)	Significant improvement in OS and PFS in refractory NSCLC	(1, 2)
Large Solid Tumors	Volume-related resistance barriers	Electro-Stress Storm (Piezo-catalysis + US)	Dismantling of deep-seated tumor resistance	(29, 30)

### 5.2 Physical induction of immune evasion: compensatory upregulation of PD-L1

Furthermore, persistent physical electro-stress can shape the evolutionary landscape of polyclonal tumor populations. By acting as an external filtering force, long-term exposure may drive progressive clonal selection, facilitating the expansion of electro-tolerant sub-clones that eventually dominate the tumor architecture (72). Beyond molecular-level resistance, the feedback regulation of tumor immune checkpoints by electric field therapy warrants careful consideration. Research in liver cancer models has explored the impact of nanosecond pulsed electric fields (nsPEF), observing that lesions surviving ablation exhibit compensatory high expression of PD-L1 (73) (**Figure 5B**). This immune escape mechanism induced by physical ablation may limit the cytotoxicity of tumor-infiltrating CD8+ T cells via the PD-1/PD-L1 signaling axis, thereby potentially leading to transient therapeutic efficacy (73) (**Table 4**). Concurrently, the feedback over-expression of programmed death-ligand 1 (PD-L1) observed in surviving cellular fractions represents an instance of microenvironmental immune and metabolic compensation, aimed at evading surveillance by effector immune cells (74). This phenomenon explores the limitations of singular electrical modalities in achieving complete tumor eradication and suggests the potential necessity of synchronously blocking immune negative regulatory pathways during physical intervention (3, 73).

This delineation of evolutionary dynamics provides a spatio-temporal reference for combination therapies. For resistance nodes categorized under transient stress and epigenetic rewiring (PARP1 and BRD4), small-molecule inhibitors may be integrated concurrently with electric fields to address the window of condition-induced vulnerability (70). Conversely, for adaptive mechanisms defined by microenvironmental compensation (e.g., PD-L1 upregulation), sequential integration with immune checkpoint inhibitors represents a potential sequence to effectively modulate systemic immune responses (75). This evolutionary oncology perspective offers an objective molecular reference for studying acquired resistance to physical interventions.

### 5.3 Precision combination strategies: reversing resistance and enhancing efficacy

Based on resistance nodes identified via multi-omics, scientists have explored several precision combination strategies to reverse therapeutic resistance:

Physical-Small Molecule Synergy: Integrating TTFields with PARP inhibitors (e.g., olaparib) or BRD4 inhibitors (e.g., JQ-1) has demonstrated significant synergistic effects in *in vitro* models, effectively inhibiting cell proliferation (6) (**Figure 5C**).

Physical-Biological Coordination: Combining oncolytic peptides such as LTX-315 or immune checkpoint inhibitors (ICIs) is considered a potential strategy to reverse electric field-induced PD-L1 upregulation, restore T cell cytotoxicity, and promote the establishment of long-term immune memory (3, 13, 73).

Multi-Physical Field Coupling: Utilizing dual-atom piezo-catalysts in conjunction with ultrasound-excited internal electric fields can generate a more intense “electro-stress storm,” exploring paths to dismantle resistance barriers in large-volume solid tumors by intensifying endoplasmic reticulum stress (29, 30) (**Figure 5D**; **Table 4**).

### 5.4 Clinical evidence: reversing acquired resistance to immunotherapy

**Figure 6:**
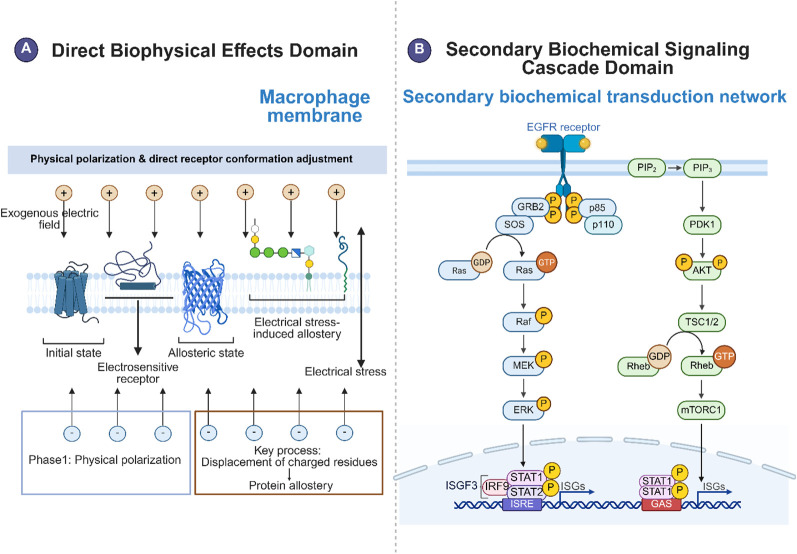
Multi-scale biophysical and biochemical dual-tier transduction network governing macrophage polarization under electrical exposure. This schematic delineates the sequential progression from exogenous physical field inputs to secondary cellular biochemical outputs. **(A)** Direct Biophysical Effects Domain (Plasma Membrane Boundary): Illustrates the immediate biophysical impacts of exogenous electric fields intersecting the macrophage plasma membrane. Physical electro-stress is suggested to force charge displacements across sensitive residues within transmembrane domains, potentially driving allosteric conformational transitions in electro-sensitive receptors. This domain focuses on the direct physical polarization and corresponding structural adjustments of membrane receptors. **(B)** Secondary Biochemical Signaling Cascade Domain (Cytoplasmic and Nuclear Compartments): Describes the downstream, cell-autonomous biochemical transduction networks mobilized indirectly by primary receptor allostery. The structural alteration of the receptor acts as an upstream signal that sequentially engages cytosolic kinase cascades, including the canonical JAK/STAT phosphorylation axis. These signaling cascades ultimately track toward the nuclear translocation of specific transcription factors, orchestrating the expression of target genes that drive the functional remodeling of macrophages into distinct phenotypic states. This stratified framework illustrates the step-by-step connection between initial physical parameters and subsequent immunological outputs.

Clinical case reports and trial data provide empirical support for these precision combination strategies (**Figure 6**). In the LUNAR trial for NSCLC and the METIS trial for brain metastases, the survival benefits of electric field therapy combined with ICIs were particularly prominent in specific subgroups (1, 2) (**Table 4**). Typical clinical cases have explored how an NSCLC patient resistant to durvalumab maintained stable disease for over 12 months after receiving PEF ablation (76). Similarly, cases of vulvar melanoma have explored how PEF induces the “abscopal effect,” leading to complete remission in lesions previously resistant to dual ICIs (16). These findings support the potential of electric field therapy as an immune sensitizer; by restructuring the tumor phenotype and immune microenvironment—including the recruitment of chemokines and regulation of macrophage polarization—it provides a viable clinical pathway for overcoming acquired immune resistance (14, 15, 22) (**Figure 6**).

## 6 Future perspectives

Research in the field of bioelectric signal transduction and immune remodeling is currently at a pivotal stage of transition from phenomenological observation to precision regulation. Based on the integration of current multi-omics landscapes, molecular dynamics simulations, and clinical evidence, future research directions may explore several dimensions with significant potential.

### 6.1 Decoding “electro-sensitive receptor” landscapes and sub-microscopic sensing mechanisms

Current understanding of electric signal sensing has reached the molecular conformational level. Future research could explore further screening of the G-protein-coupled receptor (GPCR) family regulated by direct current electric fields (dcEF), identifying more membrane proteins with similar conformational sensitivity based on findings related to NPFFR2 (5). Such molecular-level sensing mechanisms might not be limited to membrane proteins; studies could explore sub-microscopic intervention of cellular oxidative stress states at the level of electron transfer by adjusting the electronic orbital occupancy (e.g., Eg orbitals) of nanocatalysts (29). Furthermore, identifying the precise molecular nodes of cytoskeletal sensors (e.g., GEF-H1) in the transformation of physical stress into chemical signals warrants in-depth exploration as potential targets for developing novel electro-sensitizing drugs (22).

### 6.2 High-throughput closed-loop control systems and individualized physical parameter programming

To overcome the heterogeneity in electric field responses across different tumor types and individuals, the development of integrated bioelectric reactor platforms (e.g., SCHEPHERD) will be critical. Such platforms could explore large-scale automated waveform screening and closed-loop control of cellular behaviors, such as electrotaxis and polarization direction (23, 47). Future studies could explore establishing a more comprehensive “waveform parameter library” to achieve programmed management of apoptosis and pyroptosis ratios within specific microenvironments by precisely adjusting pulse width, frequency, and field strength combinations, thereby optimizing immune activation modes (12). Simultaneously, individualized simulation models (e.g., the ELLA model) incorporating anatomical features such as BMI and fat distribution will facilitate the precision delivery of physical loads in clinical settings (36).

### 6.3 Multi-omics driven dynamic combination therapy design

Electric field-induced adaptive resistance mechanisms provide a roadmap for future combination therapies. Research could explore compensatory upregulation targets such as PARP1 and BRD4 identified through proteomics to design dynamically updated dosing strategies, implementing interventions before tumors undergo resistant evolution (6). Furthermore, exploring the spatial reorganization patterns of receptors like PD-1 and MHC II induced by electric fields could facilitate the development of novel auxiliary tools to enhance immune synapse formation (15). The activation state of chemokine axes (e.g., CXCL9/10) has been proposed as a dynamic biomarker for monitoring the “cold-to-hot” transition of the microenvironment, which could be used to guide the optimal timing for immune checkpoint inhibitor (ICI) intervention (14).

## 7 Limitations

While examining the scientific value of bioelectric signal transduction and immune remodeling, it is essential to objectively scrutinize the limitations inherent in current research. These deficiencies are primarily reflected in clinical trial design, the simplification of experimental models, the direct empirical verification of molecular mechanisms, and the spatiotemporal resolution of data acquisition.

### 7.1 Ethical and methodological biases in clinical trial design

In major clinical studies conducted to date, such as the LUNAR and METIS trials, ethical considerations have necessitated an open-label design, which complicates the implementation of double-blind controls (1, 2). The absence of “sham device” controls may introduce potential bias when investigators evaluate secondary endpoints, such as radiographic progression (2). Furthermore, as some clinical studies were initiated earlier, they may not have fully integrated modern molecular profiling techniques like next-generation sequencing (NGS), to some extent limiting the in-depth interpretation of the correlation between electric field efficacy and specific genetic subtypes (1). In the assessment of neurocognitive protection, limitations in the language versions of certain testing tools have led to insufficient data completeness during multi-center follow-up (2).

Consequently, managing physical dosing amidst anatomical complexity and non-linear impedance variations remains a practical priority for clinical translation. To address this bottleneck, it is instructive to integrate lessons learned from other fields, such as non-oncological bioelectromagnetics and smart biomaterial engineering (78, 79). Within these parallel domains, investigators confronting the challenge of sustaining uniform physical loads across anisotropic matrices have developed predictive algorithms for multi-frequency impedance compensation and geometric wavefront pre-correction (80).

These cross-disciplinary experiences offer a methodological reference for managing physical tumor heterogeneity in precision electro-oncology. Future translational strategies may benefit from assimilating these control paradigms: by tracking instantaneous shifts in local electrical conductivity triggered by tissue remodeling, clinical platforms could deploy adaptive waveform adjustments and real-time phase compensation (81). This integration is suggested to help preserve a sustained physical load even within the impedance barriers characteristic of dense desmoplastic stroma, thereby optimizing downstream microenvironmental remodeling. Merging these cross-disciplinary concepts ensures that the multi-scale control narrative remains cohesively anchored to the clinical objective of treating refractory malignancies (82).

### 7.2 Challenges of model simplification and heterogeneity

The majority of current mechanistic studies on electric fields rely on highly simplified biological models. At the molecular level, although molecular dynamics simulations provide heuristic insights into receptor conformational changes (e.g., NPFFR2), these are primarily based on computational predictions and explore the current lack of direct structural biology evidence observing protein physical deformation in real-time under complex *in vivo* microenvironments (5). At the cellular level, experimental conclusions derived from single cell lines (e.g., U87) might not represent the true complexity of highly heterogeneous tumors such as glioblastoma (6). Additionally, the specific geographic or ethnic backgrounds of certain cell lines used in research warrant further validation to ensure the universality of conclusions across diverse populations (21). Regarding animal models, while subcutaneous ablation models are operationally convenient, their microenvironmental characteristics—such as the blood-brain barrier and organ-specific stroma—differ significantly from orthotopic tumors, potentially impacting the accurate assessment of immune infiltration and drug delivery efficiency (73).

Evaluating the translational potential of precision electro-oncology necessitates an objective appraisal of tissue-scale physical tumor heterogeneity and its impact on electrical delivery efficiency. Due to variations in host tissue types and internal stromal density, solid tumors exhibit non-uniform spatial dielectric profiles (72). In highly fibrotic malignancies, the deposition of extracellular matrix and dense collagen networks may function as an endogenous physical shield. This biophysical impedance can induce spatial attenuation of delivered electric fields, potentially preventing localized intensities in deep tumor cores from reaching the critical thresholds required to drive organelle stress or receptor conformational transitions, thereby limiting subsequent immune sensitization efficacy (83).

Furthermore, it is necessary to consider how tumor cell populations may cultivate acquired adaptations to the physical stimulation itself under chronic exposure. Evidence suggests that sustained physical fields may impose a selective pressure driving feedback structural remodeling. At the cytoskeletal level, tumor cells may alter microtubule polymer kinetics to support the mechanical stability of mitotic spindles against external electrodynamic forces, exhibiting potential tolerance to mitotic disruption. At the membrane level, adaptive tolerance may involve remodeling plasma membrane profiles or ion transport systems to compensate for transmembrane electrochemical potentials perturbed by physical intervention (72, 83–85). Discussing these dual physical adaptation mechanisms indicates that future paradigms may need to integrate dynamic waveform modulations to address the physical adaptation of malignant cell populations.

### 7.3 Logical gaps in molecular mechanisms and nonlinear responses

Although the activation of pathways such as cGAS-STING by electric fields has been preliminarily confirmed, certain key biochemical links remain poorly defined. For instance, the rapid degradation of STING protein observed in specific cells following electric field exposure necessitates further clarification regarding its exact molecular pathway and biological significance (11). Furthermore, in heterogeneous tissue environments, the mapping between physical parameters (e.g., pulse width, intensity) and cell death mechanisms appears non-linear; precisely guiding the dominant death mode (e.g., pyroptosis vs. apoptosis) under complex tissue architectures remains a challenge (12).

### 7.4 Insufficient spatiotemporal resolution of data acquisition

Current monitoring of immune responses often suffers from a lack of integration between “point” and “surface” data. Most immunodynamic data originate from single-point peripheral blood sampling; this spatiotemporal limitation may overlook the differential evolution of immune infiltration patterns between the tumor core and peripheral regions under electric field intervention (44). In retrospective clinical analyses, the lack of detailed records regarding specific electrode array dimensions and real-time wearing layouts limits the ability of researchers to precisely quantify the subtle contributions of different anatomical features (such as tissue depth differences caused by BMI) to survival benefits (36). These data gaps explore current constraints in the precision closed-loop optimization of physical programming protocols.

### 7.5 Bottlenecks of human clinical standardization

Although *in vitro* platforms and preclinical animal models have yielded insights into the transduction of physical stress into biological cascades, it is instructive to recognize the translational gap between these simplified systems and complex human physiology (83). Transitioning parameterized physical programming into clinical oncotherapy requires evaluating substantial biophysical and medical challenges inherent to human clinical standardization.

A primary challenge involves the complex, multi-layered dielectric heterogeneity of the human body. While electrical distributions within *in vitro* microchannels are relatively uniform, exogenous fields delivered to patients must traverse anatomically diverse strata, including skin barriers, subcutaneous adipose tissues, skeletal muscles, and dense tumor stroma. Differences in electrical conductivities and dielectric constants among these tissues can precipitate non-linear spatial attenuation of the physical load (84, 86). Ensuring that deep-seated solid tumor cores receive adequate physical intensity represents a consideration for standardizing clinical dosimetry.

Furthermore, quantitative dosimetric standardization and the management of patient compliance present practical clinical considerations. Evidence from human trials, such as the LUNAR and METIS protocols, suggests that achieving survival benefits is often associated with consistent daily device compliance over prolonged intervals (1, 2). Addressing the impact of long-term physical intervention on patients’ quality of life and exploring adaptive modulations to support compliance are relevant to the implementation of these systems.

Lastly, individual anatomical variations complicate uniform physical delivery. Recent computational simulations have demonstrated that variations in body mass index (BMI) and anatomical dimensions can influence the internal spatial positioning of delivered electric fields (36). Consequently, generalized physical prescriptions may be limited in their consistency. Future standardization may benefit from a personalized approach, incorporating patient-specific radiological metrics (such as CT or MRI scans) with computational bioelectromagnetic modeling to customize array layout geometries and evaluate individualized intervention parameters (36).

## 8 Conclusions and synthesis of available evidence

To provide an objective academic foundation for the development of precision electro-oncology, this review categorizes existing scientific evidence across hierarchical dimensions. It is instructive to distinguish between validated biophysical phenomena, emerging mechanistic insights, and trajectories driven primarily by theoretical or computational modeling, thereby maintaining scientific rigor and avoiding over-extrapolation.

### 8.1 Validated biophysical modalities and mechanisms

Current empirical evidence has established core mechanisms across multiple scales. First, intermediate-frequency alternating electric fields (e.g., TTFields) exert forces that disrupt tubulin dynamics and spindle assembly, culminating in mitotic failure (87). Second, physical electro-stress can induce localized nuclear-envelope rupture, precipitating micronuclei leakage and the subsequent activation of the cGAS-STING innate immune sensing cascade (28). Third, exceeding specific dielectric thresholds, exogenous fields induce irreversible physical electroporation damage and transmembrane ionic imbalance (88).

### 8.2 Emerging immunological and preclinical evidence

Several mechanisms possess substantial support from preclinical models but require further high-resolution validation across translational scales. One is the concept that fine-tuning physical waveform parameters (e.g., pulse width, frequency, and intensity) may modulate non-linear cell death trajectories, specifically influencing the balance between apoptosis and pyroptosis to affect DAMP release kinetics (19, 24). Another is that physical field exposure may perturb cytoskeletal equilibrium, potentially guiding the functional remodeling of tumor-associated macrophages toward highly heterogeneous spectra, such as inflammatory or interferon-responsive states (22).

### 8.3 Theoretical hypotheses requiring further validation

It is necessary to acknowledge that certain cutting-edge paradigms remain predominantly within the realm of mathematical modeling or in silico simulations, awaiting direct verification via advanced biophysical instrumentation. This includes atomistic allosteric conformational dynamic modeling of GPCRs (e.g., the NPFFR2 receptor) under physical field exposure. Constrained by simplified environments, this electro-sensitive receptor framework requires further *in situ* structural validation (5). Furthermore, conceptual strategies utilizing localized fields to dynamically modulate electronic orbital occupancy represent intriguing prospects, but require a cautious approach prior to broader translational application (52). Delineating these evidence tiers provides a structured reference for future quantitative evaluations in electro-oncology.
